# Dihydroisocoumarins, Naphthalenes, and Further Polyketides from *Aloe vera* and *A. plicatilis*: Isolation, Identification and Their 5-LOX/COX-1 Inhibiting Potency

**DOI:** 10.3390/molecules26144223

**Published:** 2021-07-12

**Authors:** Hans Wilhelm Rauwald, Ralf Maucher, Gerd Dannhardt, Kenny Kuchta

**Affiliations:** 1 Department of Pharmaceutical Biology, Leipzig University, Johannisallee 21-23, 04103 Leipzig, Germany; Ralf@Maucher.de (R.M.); kkuchta@rz.uni-leipzig.de (K.K.); 2 Department of Pharmaceutical and Medicinal Chemistry, Johannes Gutenberg-University, 55122 Mainz, Germany

**Keywords:** *Aloe vera*, *Aloe plicatilis*, dihydroisocoumarins, naphthalenes, polyketides, anti-inflammatory activity, COX-1/5-LOX

## Abstract

The present study aims at the isolation and identification of diverse phenolic polyketides from *Aloe vera* (L.) Burm.f. and *Aloe plicatilis (L.)* Miller and includes their 5-LOX/COX-1 inhibiting potency. After initial Sephadex-LH20 gel filtration and combined silica gel 60- and RP18-CC, three dihydroisocoumarins (nonaketides), four 5-methyl-8-*C*-glucosylchromones (heptaketides) from *A. vera,* and two hexaketide-naphthalenes from *A. plicatilis* have been isolated by means of HSCCC. The structures of all polyketides were elucidated by ESI-MS and 2D ^1^H/^13^C-NMR (HMQC, HMBC) techniques. The analytical/preparative separation of 3*R*-feralolide, 3′-*O*-β-d-glucopyranosyl- and the new 6-*O*-β-d-glucopyranosyl-3*R*-feralolide into their respective positional isomers are described here for the first time, including the assignment of the 3*R*-configuration in all feralolides by comparative CD spectroscopy. The chromones 7-*O*-methyl-aloesin and 7-*O*-methyl-aloeresin A were isolated for the first time from *A. vera,* together with the previously described aloesin (syn. aloeresin B) and aloeresin D. Furthermore, the new 5,6,7,8-tetrahydro-1-*O*-β-d-glucopyranosyl- 3,6*R*-dihydroxy-8*R*-methylnaphtalene was isolated from *A. plicatilis,* together with the known plicataloside. Subsequently, biological-pharmacological screening was performed to identify *Aloe* polyketides with anti-inflammatory potential in vitro. In addition to the above constituents, the anthranoids (octaketides) aloe emodin, aloin, 6′-(*E*)-p-coumaroyl-aloin A and B, and 6′-(*E*)-p-coumaroyl-7-hydroxy-8-*O*-methyl-aloin A and B were tested. In the COX-1 examination, only feralolide (10 µM) inhibited the formation of MDA by 24%, whereas the other polyketides did not display any inhibition at all. In the 5-LOX-test, all aloin-type anthranoids (10 µM) inhibited the formation of LTB_4_ by about 25–41%. Aloesin also displayed 10% inhibition at 10 µM in this in vitro setup, while the other chromones and naphthalenes did not display any activity. The present study, therefore, demonstrates the importance of low molecular phenolic polyketides for the known overall anti-inflammatory activity of *Aloe vera* preparations.

## 1. Introduction

**Figure 1:**
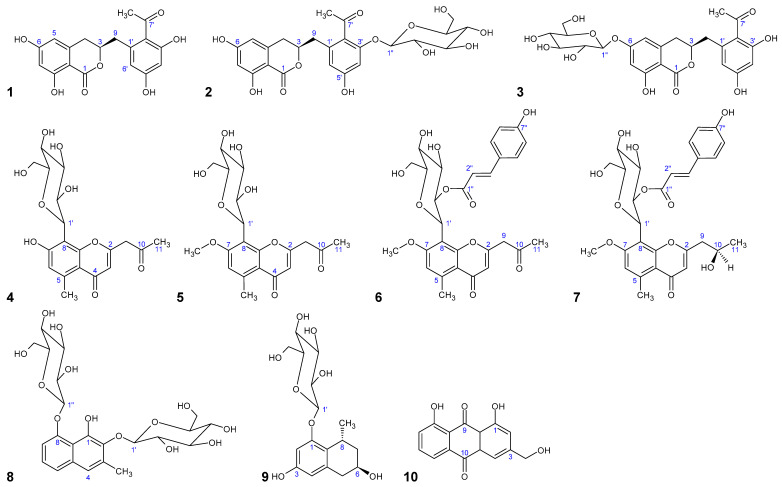

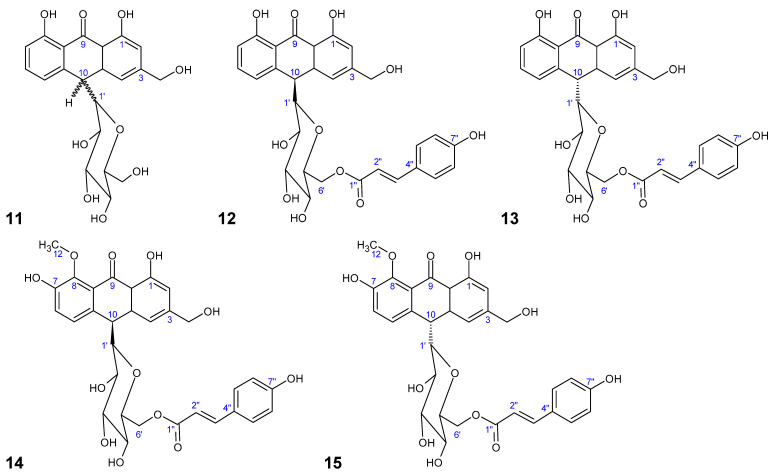
Chemical structures of isolated and tested phenolic polyketides from *Aloe vera* and *A. plicatilis*: 3*R*-feralolide (**1**); 3′-*O*-β-d-glucopyranosyl-3*R*-feralolide (**2**); 6-*O*-β-d-glucopyranosyl-3*R*-feralolide (**3**); aloesin (**4**); 7-*O*-methyl-aloesin (**5**); 7-*O*-methyl-aloeresin A (**6**); aloeresin D (**7**); plicataloside (**8**); 5,6,7,8-tetrahydro-1-*O*-β-d-glucopyranosyl-3,6-dihydroxy-8-methylnaphthalene (**9**); aloeemodin (**10**); aloins A/B (**11**); 6′-*O*-(*E*)-coumaroylaloin A (**12**); 6′-*O*-(*E*)-coumaroylaloin B (**13**); 6′-*O*-(*E*)-coumaroyl-7-hydroxy-8-*O*-methyl-aloin A (**14**); 6′-*O*-(*E*)-coumaroyl-7-hydroxy-8-*O*-methyl-aloin B (**15**).

Secondary metabolites in the genus *Aloe* are characterized by various polyketide constituents, such as naphthalenes (hexaketides), chromones (heptaketides), anthrones/anthraquinones (octaketides), and nonaketides such as feralolide [1]. Continuing our chemical investigation on polyketides from *Aloe* species [2,3,4,5,6,7], the present study aims at the isolation and identification of diverse phenolic polyketides from *Aloe vera* (L.) Burm.f. and *A. plicatilis* (L.) Miller and includes a screening of their 5-LOX/COX-1 inhibiting potency. Thus, from *A. vera,* three dihydroisocoumarins were isolated and identified, namely, 3*R*-feralolide (**1**), 3′-*O*-β-d-glucopyranosyl-3*R*-feralolide (**2**), and the new 6-*O*-β-d-glucopyranosyl-3*R*-feralolide (**3**), together with four 5-methyl-8-*C*-glucosylchromones, namely, 7-*O*-methyl-aloesin (**5**) and 7-*O*-methyl-aloeresin A (**6**), both detected for the first time in *A. vera*, with the known aloesin (**4**) and aloeresin D (**7**) (for structural formulas, see Figure 1). Furthermore, the new 5,6,7,8-tetrahydro-1-*O*-β-d-glucopyranosyl-3,6*R*-dihydroxy-8*R*-methylnaphtalene (**9**) was isolated from *A. plicatilis,* together with the known plicataloside (**8**). As *A. vera* has traditionally been used, e.g., to promote wound healing, a biological-pharmacological screening was performed to identify *Aloe* polyketides with anti-inflammatory potential in vitro. Additionally, six anthranoids isolated from *A. vera,* and previously described by our group [2,3,4,5,6,7], were tested, namely, 6′-*O*-(*E*)-coumaroyl-aloin A (**12**) and its diastereomer B (**13**), 6′-*O*-(*E*)-coumaroyl- 7-hydroxy-8-*O*-methyl-aloin A (**14**) and its diastereomer B (**15**), together with aloe emodin (**10**) and aloins A/B (**11**). All compounds were tested in an in vitro system for activity on the LTB_4_ and MDA inflammatory pathways as we previously described [8,9,10,11].

For the new tetralin derivative (**9**), obtained from *A. plicatilis*, and for the three dihydroisocoumarins, no anti-inflammatory activities have yet been reported. Speranza et al. [12] isolated feralolide (3-(2′-acetyl-5′-hydroxyphenyl)methyl-3,4-dihydro-8- hydroxy-2(1H)-benzopyran-1-on) from the *A. ferox* drug, and Veitch et al. [13] 3′-*O*-β-d-glucopyranosyl-feralolide from fresh leaves of *A. hildebrandii*. These structures, including their 3*R* configuration, were preliminarily published by our group [14,15] and recently confirmed in the flowers of *A. arborescens* [16]. Feralolide was later rediscovered in *A. vera* [17] and in *A. hijazensis* [18]. Especially strong anti-inflammatory effects were reported for aloesin derivatives [19,20,21], including the COX-2 inhibiting activity of aloesin itself [22]. Its derivatives, the two 7-*O*-methyl-aloesins (**5**, **6**), had firstly been reported from *A. rupestris* [23] and were newly found here in *A. vera*. The anthranoid octaketides (**10**–**15**) have been isolated in our lab as mentioned, and were discussed previously [2,3,4,5,6,7]. Only sporadic in vitro and in vivo experiments on the anti-inflammatory activity of single low-molecular constituents of *A. vera* leaves have been published, e.g., aloenine, aloe emodin, aloins A/B, or 2′-*O*-(*E*)-cinnamoyl-aloesinol [24]. Therefore, related polyketides might be promising leads as potential inhibitors of inflammatory pathways. Regarding used phytochemical methods, the isolation of 15 polyketides was mainly performed by means of HSCCC, after initial sephadex LH-20 gel filtration and combined silicagel-60/RP18-CC. Their structures were elucidated mainly by ESI-MS and 2d-^1^H/^13^C-NMR techniques, including HMQC and HMBC experiments, as well as CD spectroscopy.

## 2. Results and Discussion

### 2.1. Phytochemical Investigations on A. vera

The three dihydroisocoumarins, isolated for the first time from *Aloe vera* (L.) Burm.f., were identified by the following ^1^H/^13^C-NMR and ESI-MS results.

3*R*-feralolide; (3*R*-(2′-acetyl-5′-hydroxyphenyl)methyl-3,4-dihydro-8-hydroxy- 2(1H)-benzopyran-1-on) (**1**):

^1^H-NMR (MeOD, δ [ppm]): 6.31 (d, 1H, H-6′, ^4^*J*_H,H_ = 2.2 Hz); 6.27 (d, 1H, H-4′, ^4^*J*_H,H_ = 2.2 Hz); 6.22 (d, 1H, H-5, ^4^*J*_H,H_ = 2.1 Hz); 6.20 (d, 1H, H-7, ^4^*J*_H,H_ = 2.2 Hz); 4.74 (m, 1H, H-3); 3.03, 2.89 (2xm, 4H, 2xH-4, 2xH-9); 2.45 (s, 3H, 3xH-8′). ^13^C-NMR (MeOD, δ [ppm]): 205.73 (C-7′); 170.26 (C-1); 165.21 (C-6); 164.54 (C-8); 159.09 (C-3′); 160.36 (C-5′); 142.25 (C-4a); 138.30 (C-1′); 120.85 (C-2′); 110.48 (C-6′); 106.98 (C-5); 101.17 (C-7); 100.57 (C-8a); 101.47 (C-4′); 80.41 (C-3); 38.61 (C-9); 32.51 (C-4); 31.91 (C-8′). ESI-MS: 362 [M + NH_4_]^+^, 367 [M + Na]^+^, 381 [M + K]^+^.

3′-*O*-β-d-glucopyranosyl-3*R*-feralolide; (3*R*-(2′-acetyl-5′-hydroxyphenyl-3′-*O*-β-d- glucopyranosyl)methyl-3,4-dihydro-8-hydroxy-2(1H)-benzopyran-1-on) (**2**):

^1^H-NMR (DMSO-d_6_, δ [ppm]): 10.93, 9.96 (br, 2H, OH-6, OH-8); 6.53 (d, 1H, H-4′, ^4^*J*_H,H_ = 1.9 Hz); 6.41 (d, 1H, H-6′, ^4^*J*_H,H_ = 1.9 Hz); 6.18 (s(br), 1H, H-5); 6.12 (s(br), 1H, H-7); 4.86 (d, 1H, H-1″, ^3^*J*_1,2_ = 7.9 Hz); 4.55 (m, 1H, H-3); 3.69, 3.49 (m, 2H, 2xH-6″); 3.50 (m, 1H, H-5″); 3.23 (m, 1H, H-3″); 3.21 (m, 1H, H-2″); 3.19 (m, 1H, H-4″); 2.92, 2.83 (2xm, 2H, 2xH-9); 2.81, 2.80 (2xm, 2H, 2xH-4); 2.49 (s, 3H, 3xH-8′). ^13^C-NMR (DMSO-d_6_, δ [ppm]): 204.34 (C-7′); 168.60 (C-1); 163.03 (C-6); 162.56 (C-8), 158.90 (C-5′); 157.17 (C-3′); 141.75 (C-8a); 137.06 (C-1′); 120.99 (C-2′); 109.60 (C-6′); 107.21 (C-5); 99.44 (C-7); 101.75 (C-4′); 101.11 (C-1″); 99.33 (C-5a); 79.66 (C-3); 76.66 (C-3″); 75.85 (C-2″); 72.75 (C-5″); 69.33 (C-4″); 60.37 (C-6″); 37.23 (C-9); 31.91 (C-8′); 31.85 (C-4). ESI-MS: 529 [M + Na]^+^, 345 [M-Glucose]^+^.

**Figure 2:**
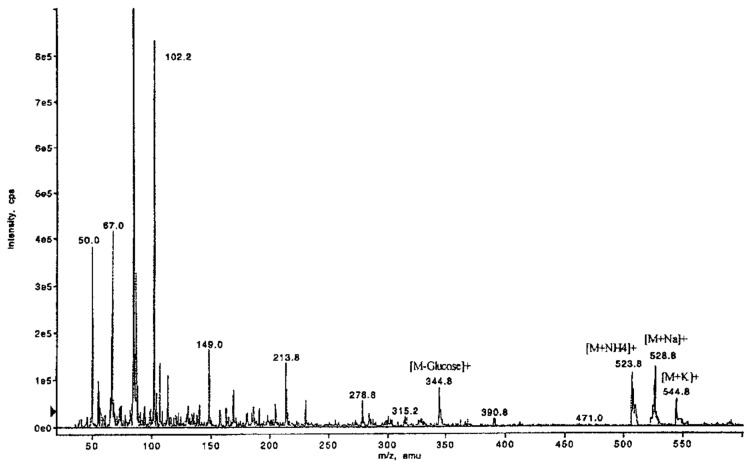
ESI-MS of 6-*O*-β-d-glucopyranosyl-feralolide (**3**). Experimental conditions: see text.

6-*O*-β-d-glucopyranosyl-3*R*-feralolide; ((3*R*)-3,4-dihydro-8-hydroxy-3-(2′-acetyl- 6-*O*-β-d-glucopyranosyl-3′,5′-dihydroxyphenyl)methyl-1H-[2]-benzopyran-1-on) (**3**):

^1^H-NMR (DMSO-d_6_, δ [ppm]): 11.04 (br, 1H, OH-8); 10.02, 9.80 (br, 2H, OH-5′, OH-3′); 6.52 (d, 1H, H-5, ^4^*J*_H,H_ = 1.9 Hz); 6.47 (d, 1H, H-7, ^4^*J*_H,H_ = 1.9 Hz); 6.30 (d, 1H, H-4′, ^4^*J*_H,H_ = 1.9 Hz); 6.23 (d, 1H, H-6′, ^4^*J*_H,H_ = 1.9 Hz); 5.33 (s(br), 1H, OH-2″); 5.10 (s(br), 1H, OH-3″); 5.04 (s(br), 1H, OH-4″); 5.00 (d, 1H, H-1″, ^3^*J*_1,2,_ = 7.9 Hz); 4.72 (m, 1H, H-3); 4.56 (s(br), 1H, OH-6″); 3.67, 3.46 (d, dd, 2H, 2xH-6″, ^3^*J*_H,H_ = 5.3 Hz, ^3^*J*_H,H_ < 1 Hz, ^3^*J*_H,H_ = 11.4 Hz); 3.39 (m, 1H, H-5″); 3.22 (dd, 1H, H-2″, ^3^J_2,3_ = 8.6 Hz); 3.27 (dd, 1H, H-3″, ^3^*J*_H,H_ = 9.0 Hz); 3.16 (dd, 1H, H-4″, ^3^*J*_H,H_ = 9.0 Hz); 2.92, 2.90 (2xm, 2H, 2xH-4); 2.97, 2.89 (2xm, 2H, 2xH-9); 2.49 (s, 3H, 3xH-8′). ^13^C-NMR (DMSO-d_6_, δ [ppm]): 203.37 (C-7′); 168.80 (C-1); 163.23 (C-6); 162.89 (C-8); 159.39 (C-5″); 157.78 (C-3″); 141.69 (C-4a); 137.00 (C-1″); 120.70 (C-2″); 109.79 (C-6″); 107.17 (C-5); 102.40 (C-8a); 101.81 (C-7); 101.37 (C-4″); 99.52 (C-1′); 79.67 (C-3); 76.39 (C-3′); 77.03 (C-5′); 73.02 (C-2′); 69.49 (C-4′); 60.53 (C-6″); 37.65 (C-9); 32.44 (C-8′); 31.91 (C-4). ESI-MS: 529 [M + Na]^+^, 345 [M-Glucose]^+^ (see Figure 2).

**Figure 3:**
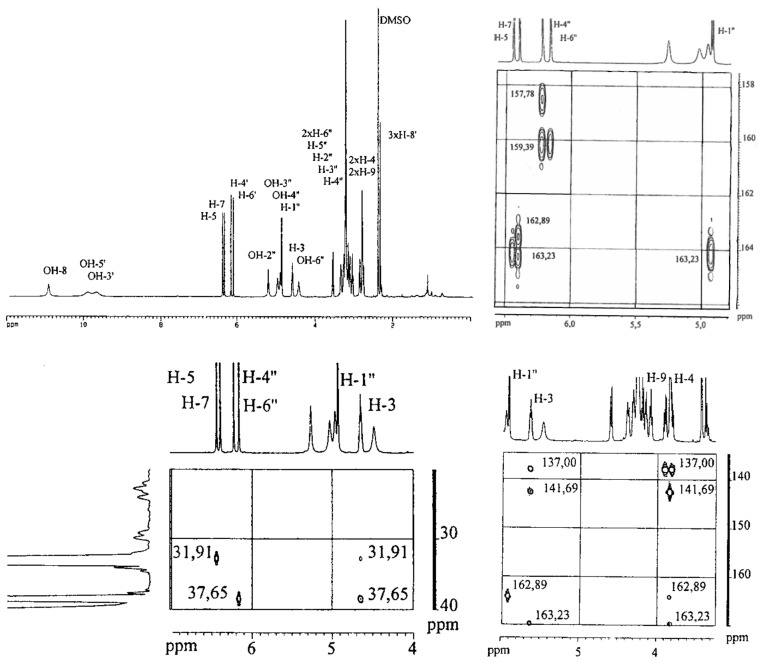
Images showing the 300 MHz-^1^H-NMR spectra of 6-*O*-β-d-glucopyranosyl-feralolide (**3**) (DMSO-d_6_), with HMBC excerpts for 20–40 ppm and 130–165 ppm.

**Figure 4:**
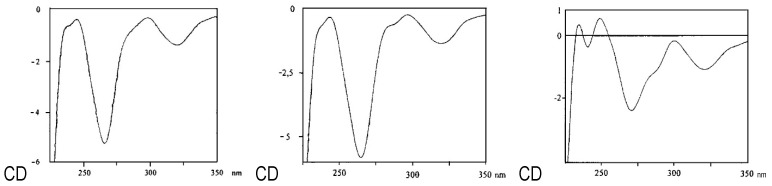
Comparison of the CD spectra of feralolide (**1**) (left), 6-*O*-β-d-glucopyranosyl-feralolide (**3**) (center) and 3′-*O*-β-d-glucopyranosyl-feralolide (**2**) (right) for configurational 3*R* determination.

**Figure 5:**
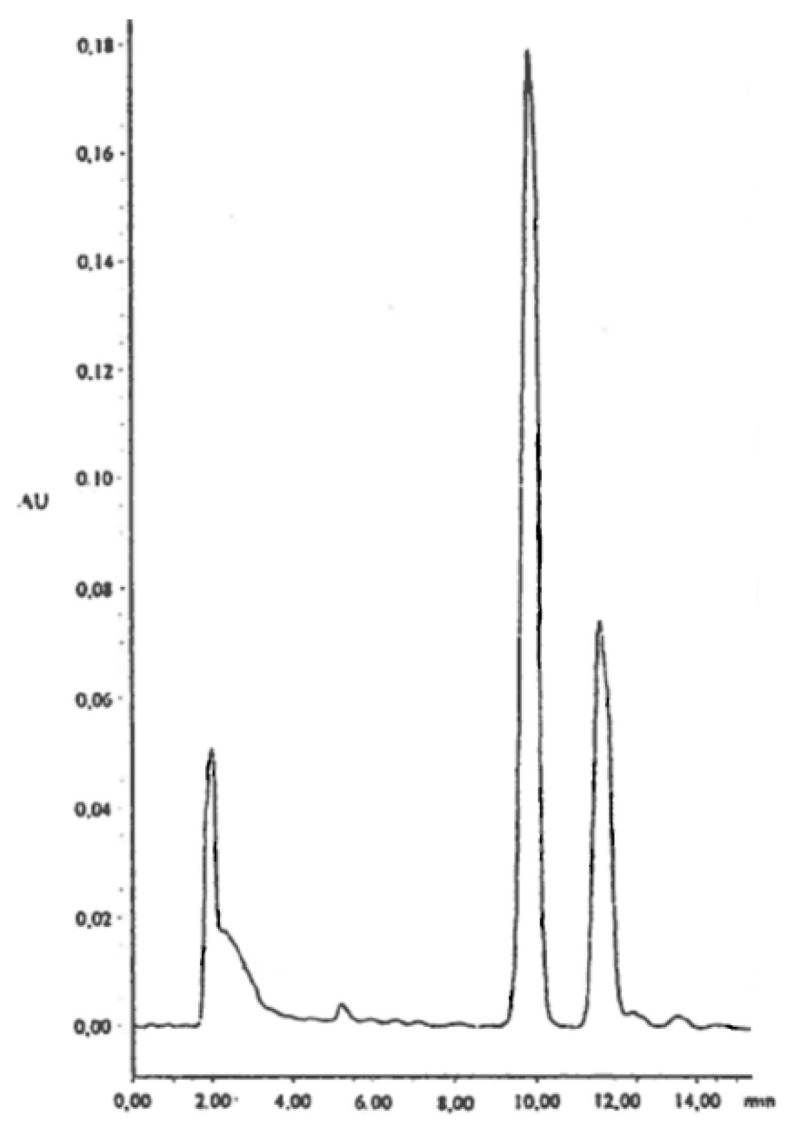
Semipreparative HPLC separation (for conditions, see text) with resolution of the positional isomers 6-*O*-β-d-glucopyranosyl-feralolide (**3**) (t_R_ = 10 min) and 3′-*O*-β-d-glucopyranosyl-feralolide (**2**) (t_R_ = 12 min) relative to their approximately 2.5:1 ratio in the *A. vera* drug is here reported for the first time.

These experimental data are in good accordance with those published for the first isolation of feralolide (**1**) from *A. ferox* [12] and could be further validated by additional NOE measurements. The structure of 3′-*O*-β-d-glucopyranosyl-feralolide (**2**) was identified by the above given NMR data, in comparison with the literature [13] and additional NOE measurements, whereby the attachment of the glucosyl moiety at C3′-OH could be verified for the first time in the present study. The structure of the new 6-*O*-β-d-glucopyranosyl-feralolide (**3**) was identified by the above-given NMR, including HMBC data and additional NOE measurements. In contrast to 3′-*O*-β-d-glucopyranosyl-feralolide (**2**), the attachment of the glucosyl moiety at C6-OH was determined by the corresponding HMBC and NOE data (see Figure 3 and Figure 4). All feralolides (**1**, **2**, and **3**) show identical negative and positive signs in the comparison of their CD spectra, thus enabling a clear identification of their 3*R* configuration (Figure 4). In terms of quantity, aglycone **1** is the major feralolide in the *A. vera* drugs, while glucosides **3** and **2** are present in a ratio of 2.5:1, as shown by HPLC separation (Figure 5).

In detail, the chromatographic and spectroscopic data of (**2)** and (**3)** displayed a high degree of similarity. The only significant difference between these two substances is the cross peak between the carbon atom C6 and the anomeric H1″ in the HMBC spectrum in the latter compound, which proves the bond of the glucose moiety to the C6 carbon. The protons at C5 and C7 exhibit cross peaks with the carbons C6 and C6 & C8, respectively. The protons at C4′ and C6′ display cross peaks with the carbons C3′ & C5′ and C5′, respectively (Figure 3). Further cross peaks observed in the HMBC spectrum are those between the protons at C5 and C6′ and the carbons C4 and C9, respectively, as well as between the proton C3 and the carbons C4 & C9 (Figure 3). The methylene groups of C4 and C9 display further cross peaks with the carbons C1′, C1, C4a (between C4 and C5), and C6. Finally, three further cross peaks appear between H3 and the carbon atoms C1′, C1, and C4a (Figure 3). Judging both from the ESI-MS (Figure 2) and NMR data, the aglycone of 6-O-β-D-glucopyranosyl-feralolide (**3**) could clearly be identified as feralolide. However, in contrast to 3′-*O*-β-d-glucopyranosyl-feralolide (**2**), the glucose moiety was demonstrated to be attached to the 6-position as an *O*-glycoside, as strongly hinted by the low-field shift of about 2 ppm observed for the ^13^C-NMR values at C3′ of 3′-*O*-β-d-glucopyranosyl-feralolide and C6 of 6-*O*-β-d-glucopyranosyl- feralolide, respectively. As far as the stereochemistry at C3 and C9 is concerned, the measured CD data are clearly identical to those of feralolide (Figure 4), proving all three molecules to exhibit an *R*-configuration. Therefore, (3*R*)-6-*O*-β-d-glucopyranosyl- feralolide (**3**) was firstly isolated from *Aloe* spp. as a new natural product in the study at hand. As far as 3′-*O*-β-d-glucopyranosyl-feralolide (**2**) is concerned, its steric structure at the C3 carbon had not been reported when it was first discovered in *A. hildebrandii* by [13], and has now been established as 3*R*, recently independently confirmed from flowers of *A. arborescens* [16]. Surprisingly, feralolide (**1**) was recently found in *A. vera* gel in two Yemeni *Aloe* spp. by LC-MS [25], confirming our previous assumption from TLC experiments [14,15] that dihydroisocoumarins are also gel constituents.

**Figure 6:**
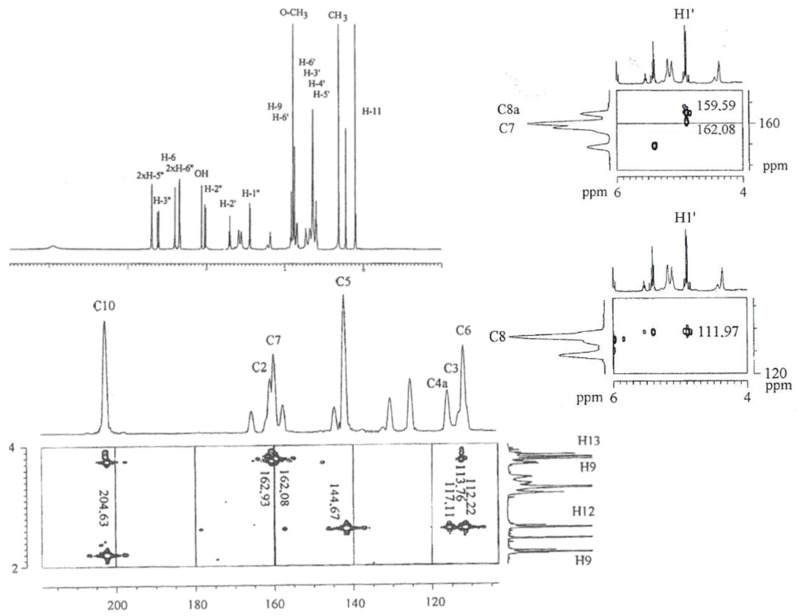
Images showing the 300 MHz-^1^H- and ^13^C-NMR spectra of 7-*O*-methyl-aloeresin A (**6**), with HMBC excerpts for 155–170 ppm, 105–120 ppm and 100–215 ppm.

Regarding aloesin (**4**) and aloesin-type chromones in *A. vera*, 7-O-methyl-aloesin (**5**) and its 2′-*O*-p-coumaroyl-derivative 7-*O*-methyl-aloeresin A (**6**) have been isolated for the first time from this plant, besides aloeresin D (**7**), which was previously isolated by our group [3]. The occurrence of **5** and **6** was first described in *Aloe marlothii* A.Berger and *Aloe rupestris* Baker [23]. Their structures were investigated by 2d-NMR, HMBC, HMQC, and COSY, as well as ESI-MS, in our laboratory [14,15] (Figure 6). 

7-*O*-methyl-aloesin; (2-acetonyl-8-*C*-β-d-glucopyranosyl-7-methoxy-5-methylchro- mone) (**5**):

^1^H-NMR (DMSO-d_6_, δ [ppm]): 6.93 (s, 1H, H-6); 6.13 (s, 1H, H-3); 4.69 (d, 1H, H-1′, ^3^*J*_H,H_ = 10.1 Hz); 3.88 (s, 3H, O-CH_3_); 3.76 (dd, 1H, H-2′, ^3^*J*_H,H_ = 10.1 Hz); 3.72 (m, 2H, H-9); 3.68, 3.36 (2xm, 2H, 2xH-6′); 3.19 (m, 1H, H-5′); 3.13 (m, 1H, H-3′); 3.09 (m, 1H, H-4′); 2.74 (s, 3H, CH_3_); 2.21 (s, 3H, 3xH-11). ^13^C-NMR (DMSO-d_6_, δ [ppm]): 204.48 (C-10); 182.10 (C-4); 162.93 (C-2); 162.70 (C-7); 159.28 (C-8a); 144.13 (C-5); 114.06 (C-4a); 113.43 (C-3); 113.36 (C-8); 113.09 (C-6); 82.50 (C-3′); 80.16 (C-5′); 74.67 (C-1′); 72.80 (C-2′); 72.07 (C-4′); 63.28 (C-6′); 56.94 (O-CH_3_); 49.60 (C-9); 30.01 (C-11); 23.64 (CH_3_). ESI-MS: 431 [M + Na]^+^

7-*O*-methyl-aloeresin A; (2-acetonyl-8-*C*-β-d-(2-*O*-(*E*)-p-coumaroyl)glucopyranosyl- 7-methoxy-5-methylchromone) (**6**):

^1^H-NMR (DMSO-d_6_, δ [ppm]): 9.90 (s(br), 1H, OH-7″); 7.44 (d, 2H, 2xH-5″, ^3^*J*_H,H_ = 8.5 Hz); 7.28 (d, 1H, H-3″, ^3^*J*_H,H_ = 15.7 Hz); 6.85 (s, 1H, H-6); 6.74 (d, 2H, 2xH-6″, ^3^*J*_H,H_ = 8.5 Hz); 6.19 (s, 1H, H-3); 6.05 (d, 1H, H-2″, ^3^*J*_H,H_ = 15.7 Hz); 5.46 (dd, 1H, H-2′, ^3^*J*_H,H_ = 8.9 Hz); 5.23 (s(br), 1H, OH-3′); 5.17 (s(br), 1H, OH-4′); 4.94 (d, 1H, H-1′, ^3^*J*_H,H_ = 10.1 Hz); 4.42 (s(br), 1H, OH-6′); 3.84 (s, 3H, O-CH_3_); 3.80 (s(br), 2H, H-9); 3.74, 3.41 (2xm, 2H, 2xH-6′); 3.51 (m, 1H, H-3′); 3.25 (m, 1H, H-4′); 3.34 (m, 1H, H-5′); 2.76 (s, 3H, CH_3_); 2.26 (s, 3H, H-11). ^13^C-NMR (DMSO-d_6_, δ [ppm]): 204.63 (C-10); 182.06 (C-4); 168.06 (C-1″); 162.93 (C-2); 162.08 (C-7); 161.25 (C-7″); 159.59 (C-8a); 145.25 (C-3″); 144.67 (C-5); 130.81 (C-5″); 127.00 (C-4″); 117.11 (C-4a); 116.66 (C-6″); 115.11 (C-2″); 113.76 (C-3); 112.22 (C-6); 111.97 (C-8); 82.80 (C-5′); 77.81 (C-3′); 73.89 (C-2′); 72.53 (C-4′); 72.14 (C-1′); 63.33 (C-6′); 57.12 (O-CH_3_); 49.10 (C-9); 29.89 (C-11); 23.67 (CH_3_). ESI-MS: 555 [M + H]^+^

On the subject of the octaketides, the diastereomeric anthrone-*C*-glucosyl compounds of the aloin-type included in this biological screening have been preliminarily published [14,15], namely, 6′-*O*-(*E*)-coumaroyl-aloin A (**12**) and its B-diastereomer (**13**), 6′-*O*-(*E*)-coumaroyl-7-hydroxy-8-*O*-methyl-aloin A (**14**), and its B-diastereomer (**15**), as well as aloe emodin (**10**) and aloins A/B (**11**).

### 2.2. Phytochemical Investigations on A. plicatilis

**Figure 7:**
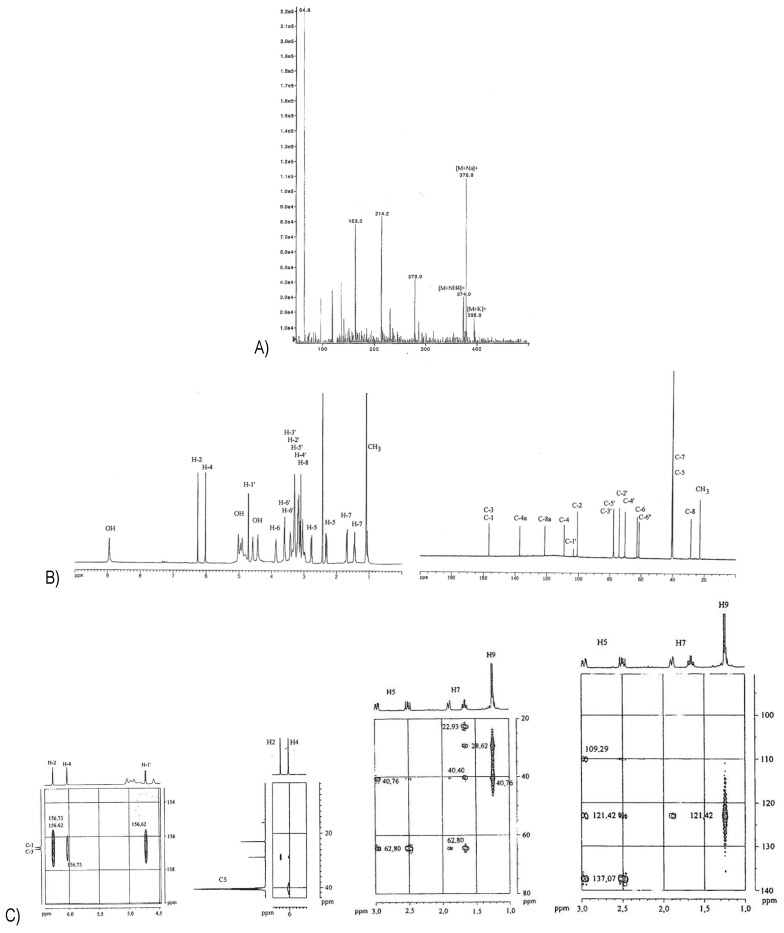

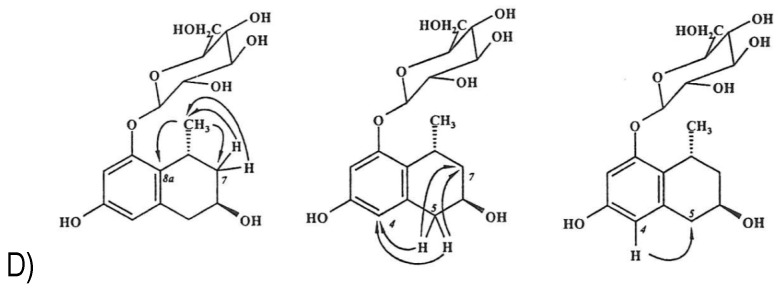
(**A**) ESI-MS of **9**; (**B**) ^1^H- (left) and ^13^C-NMR spectra (right) of **9**; (**C**) excerpts from its HMBC spectrum; (**D**) couplings in **9** as presented in the HMBC spectrum.

**Figure 8:**
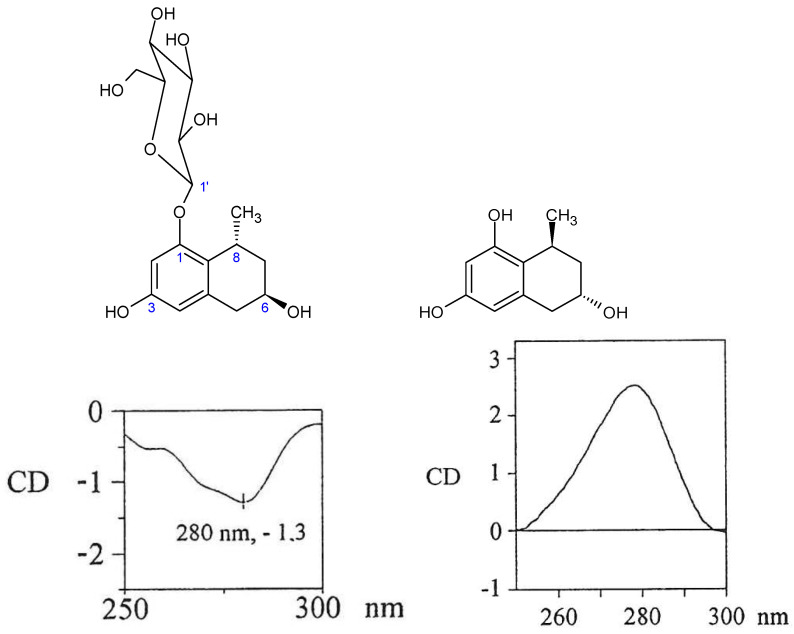
Opposite signs at 280 nm in the CD of **9** (**left**) and feroxidin (**right**) [27,28].

A methanol-water extract from freshly collected leaves of *A. plicatilis* was fractionated by combined silica gel and sephadex-LH20 CC, yielding the naphthalene derivatives **8** and **9**. Derivative **8** was identified as the known substituted naphthalene diglucoside plicataloside [26]. Compound **9** was identified as the novel 1-methyltetralin derivative 5,6,7,8-tetrahydro-1-*O*-β-d-glucopyranosyl-3,6-dihydroxy-8-methylnaphtha- lene by the NMR data and additional NOE measurements. Three 1-methyltetralins were previously isolated from commercial Cape aloes. First, the aglycone feroxidin was characterized as 3,6,8-trihydroxy-1-methyltetralin with a 6S, 8S configuration [27,28]. Subsequently, the 3-O-glucoside (feroxin A), and its p-coumaric acid ester (feroxin B), were described [29]. Surprisingly, despite identical molecular masses, our isolated compound **9** from *A. plicatilis* is also a 1-methyltetralin derivative, but with a 6*R*, 8*R* configuration. Extensive ^1^H/^13^C-NMR experiments (Figure 7) and CD spectra with opposite signs (e.g., at 280 nm; see Figure 8) to the feroxins clearly demonstrate that **9** displays the opposite sterical configuration, as compared to feroxin A. Additionally, the glucosylation has not taken place at the alcoholic hydroxyl at C6, as in feroxin A, but at the phenolic 1-OH, as also demonstrated by various NMR measurements. 

5,6,7,8-tetrahydro-1-*O*-β-d-glucopyranosyl-3,6-dihydroxy-8-methylnaphthalene (**9**):

^1^H-NMR (DMSO-d_6_, δ [ppm]): 8.93 (s, 1H, OH-3); 6.34 (s, 1H, H-2); 6.11 (s, 1H, H-4), 5.11 (s(br), 1H, OH-2′); 5.04 (s(br), 1H, OH-3′); 4.98 (s(br), 1H, OH-4′); 4.78 (d, 1H, H-1′, ^3^*J*_H,H_ = 7.5 Hz); 4.66 (s(br), 1H, OH-6); 4.51 (s(br), 1H, OH-6′); 3.94 (m, 1H, H-6); 3.68, 3.50 (2xd, 2H, 2xH-6′, ^3^*J*_H,H_ = 11.24 Hz); 3.29 (m, 1H, H-3′); 3.26 (m, 1H, H-2′); 3.24 (m, 1H, H-5′); 3.21 (s, 1H, H-4′); 3.12 (m, 1H, H-8); 2.85, 2.40 (2xdd, 2H, 2xH-5, ^3^*J*_H,H_ = 10.34 Hz); 1.76 (d, 1H, H-7, ^3^*J*_H,H_ = 11.77 Hz); 1.52 (m, 1H, H-7); 1.16 (d, 3H, CH_3_, ^3^*J*_H,H_ = 6.93 Hz). ^13^C-NMR (DMSO-d_6_, δ [ppm]): 156.73 (C-3); 156.62 (C-1); 137.07 (C-4a); 121.42 (C-8a); 109.29 (C-4); 101.03 (C-1′); 100.97 (C-2); 77.91 (C-3′); 77.77 (C-5′); 74.26 (C-2′); 70.50 (C-4′); 62.80 (C-6); 61.50 (C-6′); 40.76 (C-7); 40.40 (C-5); 28.62 (C-8); 22.93 (CH_3_). ESI-MS: 374 [M + NH_4_]^+^, 379 [M + Na]^+^, 395 [M + K]^+^.

### 2.3. Biological Screening of 15 Polyketides on Their 5-LOX/COX-1 Inhibiting Potency

**Figure 9:**
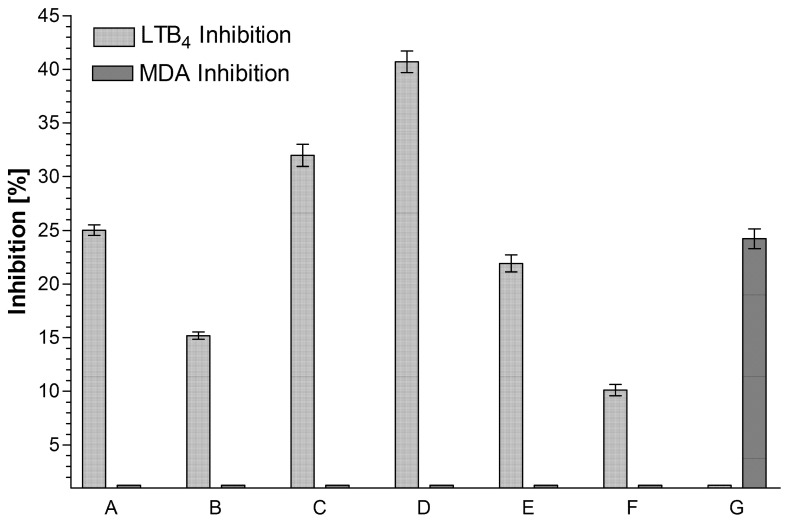
Inhibiting effect of the isolated *Aloe* polyketide constituents on the biosynthesis of the inflammation mediators LTB_4_ and MDA in the above described in vitro test systems. Among these, only those that did display activity in at least one of the two test systems are depicted. All measurements were performed in triplicates at a concentration of 10 µM for all tested substances (for further details, see Materials and Methods). (**A**) aloin A/B (25.00% LTB_4_ inhibition) (**11** in Figure 1); (**B**) aloe emodin (15.00% LTB_4_ inhibition) (**10** in Figure 1); (**C**) 6′-*O*-(*E*)-coumaroyl-aloin A (32.00% LTB_4_ inhibition) (**12** in Figure 1); (**D**) 6′-*O*-(*E*)-coumaroyl-aloin B (41.00% LTB_4_ inhibition) (**13** in Figure 1); (**E**) 6′-*O*-(*E*)-coumaroyl-7-hydroxy-8-*O*-methyl-aloin B (22.00% LTB_4_ inhibition) (**15** in Figure 1); (**F**) aloesin (10.00% LTB_4_ inhibition) (**4** in Figure 1); (**G**) 3*R*-feralolide (24.00% MDA inhibition) (**1** in Figure 1).

In the applied 5-LOX screening system [11] the aloin/anthraquinone derivatives (at a concentration of 10 µM) displayed inhibition activities between 25 and 41% of LTB_4_ production. Among the tested chromones, only aloesin (**4**) displayed any inhibitory activity that was also limited to the LOX system. All tested naphthalene derivatives were inactive in both applied test systems. Among the tested substances, only feralolide (**1**) (at a concentration of 10 µM) achieved a 24% reduction of MDA production in the COX-1 test. All other tested substances were inactive in this test system. Feralolide did not, however, show any effect on the production of LTB_4_. Even if the glycosylated feralolide derivatives were inactive in the applied test system, they may very well be active in the human body, as the function of glycosides as inactive prodrugs that are transformed into active components by the human metabolism after ingestion is a well-documented fact in pharmacokinetics [30]. The anthraquinones emodin and aloe emodin (**10**) have already been identified as lipoxygenase inhibitors [31]. In particular, the individual findings of the anti-inflammatory activity of the chromones are obvious, especially under consideration of the described COX-2 inhibition by aloesin (**4**) itself [22], in contrast to the COX-1 inhibition observed here (Figure 9). Possibly, a desired dual inhibition of COX-2 and 5-LOX may be a new strategy to provide safer non-steroidal anti-inflammatory drugs. Consequently, equivalent studies should be carried out for many previously reported anti-inflammatory chromones, including free and esterified aloesins [18,19,20,21,32].

Most of these plant polyketides have been isolated, together with related isocoumarins from the Japanese medicinal and recreational tea plant, amacha, meaning “sweet tea” in Japanese (*Hydrangea macrophylla* Seringe var. *thunbergii* Makino) [33,34], the naturally sweet taste of which is caused by the dihydroisocoumarin phyllodulcin, with a sweetening effect that is 400–800 times sweeter than sugar [35]. In accordance with its application in Japanese Kampo medicine, several other dihydroisocoumarins from this source—namely, hydrangenol, hydrangenol-8-*O*-glucoside, (−)-hydrangenol-4′-*O*-glucoside, thunberginol C, thunberginol D, thunberginol E, and thunberginol G—have been described to exert substantial anti-allergic and antihistaminic effects [36,37]. Furthermore, its dihydroisocoumarins, 3*R*-phyllodulcin, thunberginol C, thunberginol D, and thunberginol G, have been found to display differentiation-inducing activities against leukemic cells [38], hinting at a potential application for cancer therapy. The same is true for the dihydroisocoumarin (3*R*,4*R*)-(−)-6-methoxy-1-oxo-3-pentyl-3,4-dihydro-1H-isochromen-4-yl-acetate, isolated from the Brazilian medicinal plant *Xyris pterygoblephara* Steud., which showed strong aromatase inhibitory activity in an in vitro breast cancer model [39]. In the traditional medicine of Brazil, this plant is mainly used for diverse dermatological indications, which corresponds to the reported activity of its dihydroisocoumarin (3*R*,4*R*)-(−)-6- methoxy-3,4-dihydro-3-n-pentil-4-acethoxy-1H-2-benzopyran-1-one against dermato- parasitic fungi [40]. Most recently, the isolation of a dihydroisocoumarin with activity on the GABA A neuronal receptor from the Moroccan herb *Haloxylon scoparium* Pomel has also been reported [41].

The documented reduction of MDA production in the COX-1 test by feralolide constitutes the first attribution of biological activity to a dihydroisocoumarin of an *Aloe* species. The presented proof of its anti-inflammatory activity is of high relevance in the context of the typical application of *A. vera* in wound healing and a variety of inflammatory diseases, all of which have been validated by successful clinical studies [42,43,44,45] and even meta-analysis [46]. Feralolide might be of especial interest in these dermatological indications, as similar dihydroisocoumarins have been identified as active constituents of Japanese amacha (meaning “sweet tea”) (*Hydrangea macrophylla* Seringe var. *thunbergii* Makino) [36,37] and the Brazilian *Xyris pterygoblephara* Steud. [40], both of which are traditionally used against skin inflammations. Although the *Aloe* spp. dihydroisocoumarins were isolated from the laxative aloe resin drug in the present research project, there is no reason to assume that the presence of these compounds in the living plants should be limited to the resin-storing vessels as described above. They may therefore very well be of high interest in the context of the numerous dermatological applications of the *Aloe* spp. gel drugs. Furthermore, anticancer activities reported from Brazilian traditional medicine for dihydroisocoumarins in the literature [38,39] might also be of interest for the feralolide derivatives of *Aloe* spp., as the use of whole leaf macerates of *Aloe arborescens* Mill. as an anticancer drug is one of its most famous applications in this ethnopharmacological tradition [47]. However, how much dihydroisocoumarins might really contribute to this effect has to be clarified in further, independent experiments.

## 3. Material and Methods

### 3.1. Isolation of Pure Dihydroisocoumarins (***1***, ***2***, and ***3***)

*Aloe barbadensis* (syn. *A. vera*) drug (Ph.Eur.) (Lot No. Tot.J/W27.02.84/038) was obtained from Müggenburg Pflanzliche Rohstoffe (Bad Bramstedt, Germany). For the analysis, 100 g of the powdered drug were extracted with 2 l of a mixture of EtOAc/H_2_O (9:1, *v*/*v*) under constant shaking for 17 h. After filtration through a frit for removing insoluble components, the organic and aqueous phases were separated. Afterward, the organic phase was extracted again, first with 100 mL of water, then with 100 mL of a saturated aqueous NaCl solution, and then once again with 100 mL of water. Finally, the organic phase was dried over Na_2_SO_4_ and subsequently evaporated to dryness under reduced pressure, resulting in 22.8 g of dry extract. A part of the organic fraction (20 g) was adsorbed to 80 g of silica gel (40–63 µm) and subjected to normal phase silica gel CC (2.1 kg, CHCl_3_–MeOH (3:1, *v*/*v*)) to give nine fractions (Fr.A1 (2.2 g), Fr.A2 (3.5 g), Fr.A3 (4.6 g), Fr.A4 (7.2 g), Fr.A5 (0.8 g), Fr.A6 (0.7 g), Fr.A7 (0.4 g), Fr.A8 (0.3 g), Fr.A9 (0.1 g)). The entire 2.2 g of Fr.A1 were subjected to HSCCC (MKII CCC, Zinsser Analytics, Frankfurt, Germany) (stationary phase: organic; mobile phase: aqueous; (CHCl_3_/MeOH/H_2_O) 7:13:8; 1 mL/min) to give three fractions (Fr.B1 (1.22 g), Fr.B2 (0.75 g), Fr.B3 (0.58 g)). After this, 255 mg of Fr.B2 were subsequently adsorbed to 0.4 g of silica gel (40–63 µm) and subjected to normal phase silica gel CC (35 g, CHCl_3_–MeOH (3:1, *v*/*v*)), eluting feralolide (**1**) (114 mg) as a pure compound. Fr.A5 and Fr.A6 were pooled, adsorbed to 5 g of silica gel (63–200 µm) and subjected to normal phase silica gel CC (35 g, EtOAc → EtOAc–MeOH (100 → 100:5, *v*/*v*)) to give seven fractions (Fr.C1 (95 mg), Fr.C2 (180 mg), Fr.C3 (80 mg), Fr.C4 (210 mg), Fr.C5 (120 mg), Fr.C6 (350 mg), Fr.C7 (450 mg)). Fr.A5 and Fr.A6 were pooled and the entire 800 mg subjected to HSCCC (stationary phase: organic; mobile phase: aqueous; (CHCl_3_/MeOH/H_2_O) 7:13:8; 2 mL/min) to give three fractions (Fr.D1 (235 mg), Fr.D2 (90 mg), Fr.D3 (198 mg)). In a final step, fraction Fr.D2 was purified by gradient semipreparative HPLC (Waters 600 Controller and Pump, Waters Tunable Absorbance Detector Model 486) (Nucleosil C18 (Macherey-Nagel, Düren, Germany); H_2_O–MeOH (6:4 → 4:6) 30 min), 6-*O*-β-d-glucopyranosyl-feralolide (**3**) (36 mg) and 3′-*O*-β-d-glucopyranosyl-feralolide (**2**) (13 mg) as pure compounds, at Rt = 15 min and Rt = 19 min, respectively.

### 3.2. TLC Examination

All extract fractions in the above-described isolation protocol were controlled for the presence of desirable substances using silica gel 60 F_254_ TCL plates (Merck, Darmstadt, Germany). Depending on the chromatographic properties of the extract fractions encountered during the isolation procedure, either (EtOAc/EtOH/H_2_O; 100:20:13) or (CHCl_3_/MeOH/H_2_O/HCOOH; 70:130:80:1) was used as mobile phase A and B, respectively. Detection took place under UV light (quenching at 254 nm and fluorescence at 366 nm) and in the daylight after derivatization with either 5% methanolic KOH (after incubation at 105 °C for 10 min) or with a 0.5% aqueous solution of fast blue B zinc salt (di-o-anisidine diazotatezinc double salt), depending on the properties of the individual extract. When using mobile phase A, **1**, **2**, and **3** displayed R_f_ values of 0.96, 0.66, and 0.63, while the corresponding values for mobile phase B were 0.69, 0.32, and 0.33, respectively.

### 3.3. ESI-MS, NMR and CD Spectra

As far as the applied NMR instruments are concerned, a Varian (Palo Alto, CA, USA) Gemini 300 Spectrometer (^1^H: 300.075 MHz, ^13^C: 75.462 MHz), a Bruker (Rheinstetten, Germany) Advance DRX-400 Spectrometer (^1^H: 399.952 MHz, ^13^C: 100.577 MHz, HMQC: 160 MHz, HMBC: 8 MHz), and a Bruker DRX-600 Spectrometer (^1^H: 600.133 MHz, ^13^C: 150.918 MHz, HMQC: 160 MHz, HMBC: 8 MHz) were used. An internal reference relative to the respective solvent system was used as a standard. The structures of all three isolated compounds were additionally identified via Nuclear Overhauser Effect (NOE) experiments. All mass spectra were recorded using a Sciex 365 API No. 99 MS-MS instrument from Perkin-Elmer (Waltham, MA, USA) in electrospray ionization mode. CD spectra were recorded on a Jasco715-spectrometer (Pfungstadt, Germany).

### 3.4. Isolation of 5,6,7,8-Tetrahydro-1-O-β-D-glucopyranosyl-3,6-dihydroxy-8-methylnaphthalene (***9***) from Aloe plicatilis 

Fresh leaves of *Aloe plicatilis* were obtained from the Palmengarten in Frankfurt am Main. For isolation, 242.5 g of the leaves were cut into cubes of 2 cm diameter and homogenized in an Ultra Turrax T50 for 1h under cooling with ice in 500 mL methanol. After filtration, the resulting extract was evaporated to dryness in a vacuum (fraction A, 4.3 g). The remaining plant material is homogenized for 1 h with 250 mL water and 250 mL methanol. After filtration, the resulting extract is evaporated to dryness in a vacuum (fraction B, 1.6 g). Then, 4.2 g of fraction A were suspended in 50 mL methanol. After removing the insoluble parts, the liquid extract was evaporated to dryness in a vacuum, yielding 2.44 g of dry extract. This extract was divided into two equal portions and separated using two identical CC setups (column A: silica gel 40–63 µm, liquid phase: EtOAc 100 mL, EtOAc/MeOH (10:1) 200 mL, EtOAc/MeOH (10:2) 200 mL, EtOAc/MeOH (10:3) 200 mL, EtOAc/MeOH (10:4) 200 mL). During this CC, two fractions could be identified via TLC detection, namely, fraction 1 (0.13 g/R_f_ (SiO_2_): 0.47 (EtOAc/MeOH/H_2_O 100:17:13)) and fraction 2 (0.25 g/R_f_ (SiO_2_): 0.11 (EtOAc/MeOH/H_2_O 100:17:13)). Subsequently, plicataloside (**8**) (0.12 g/R_f_ (SiO_2_): 0.09 (EtOAc/MeOH/H_2_O 100:20:13)/0.15 (CH_3_Cl_3_/MeOH/H_2_O/HCOOH 7:13:8:0.1)) was isolated via CC of 0.24 g of fraction 2 with Sephadex LH 20 and methanol as a mobile phase, as a pure, solid yellow substance. Likewise, 5,6,7,8-tetrahydro-1-*O*-β-d-glucopyranosyl-3,6-dihydroxy-8- methylnaphthalene (**9**) (58 mg/R_f_ (SiO_2_): 0.59 (EtOAc/EtOH/H_2_O 100:20:13)/0.19 (CH_3_Cl_3_/MeOH/H_2_O/HCOOH 7:13:8:0.1)) was isolated via CC of 0.11 g of fraction 1 on Sephadex LH 20 and methanol as a mobile phase, and also as a pure, solid yellow substance.

### 3.5. In Vitro Screening for Anti-Inflammatory Activity by Inhibiting COX-1 (MDA Assay)

During the catalysis of PGH_2_ (prostaglandin H2) into TXA_2_ (thromboxane A2) by the thromboxane synthase system, malondialdehyde (MDA) is produced at a ratio of 1:1 with 12-HHT (12(S)-hydroxyheptadeca-5Z, 8E, 10E-trienoic acid). MDA can be detected photometrically after aldol condensation with thiobutyric acid [48]. For the in vitro screening, 1.5 l of pig blood was collected in a plastic bucket that already contained 150 mL of sodium-EDTA solution (0.077 M), immediately after sacrificing the animal. The blood was diluted with 700 mL of isotonic PBS (phosphate-buffered saline) solution and centrifuged for 20 min at 200× *g.* The supernatant, which is rich in thrombocytes, was carefully collected using a syringe and centrifuged once again for 20 min at 200× *g* to remove all remaining erythrocytes. In the subsequent centrifugation step at 1000× *g* for 15 min, the thrombocytes sediment and can finally be re-suspended in isotonic PBS at a defined cell density of 10^9^ cells/mL. All isolation steps can be performed in plastic vessels at room temperature. For the test, 700 µL of this thrombocyte suspension was placed in 1.5 mL Eppendorf vessels (Eppendorf AG, Hamburg, Germany), together with 10 µL of the respective inhibitor solution, and incubated for 10 min at 37 °C. The enzyme reaction was started by adding 50 µL of calcium ionophore buffer (final concentration: 5 µM) and terminated after 10 min at 37 °C by adding 400 µL of trichloroacetic acid. All samples were centrifuged at 4000× *g* for 15 min, after which the supernatant was used for determining the MDA concentration. A negative control was performed in four repetitions, in which only 10 µL of DMSO solvent were added to the reaction mixture instead of the respective isolated compounds. For the positive control (*n* = 2), 700 µL of thrombocyte suspension was mixed with 10 µL of solvent and incubated at 37 °C. After 10 min, 400 µL of trichloroacetic acid and subsequently 50 µL of calcium ionophore buffer were added to the mixture. The subsequent steps were identical to those described for the extracted *Aloe* constituents, as described above. For determining the MDA concentration, 0.5 mL of the abovementioned supernatant was mixed with 0.5 mL of thiobarbituric acid solution. This mixture was incubated in a water bath for 30 min at 70 °C. After another 30 min of incubation at room temperature, the amount of MDA was measured in a spectral fluorometer (λ-excitation: 533 nm, λ-emission: 550 nm).

### 3.6. In Vitro Screening for Anti-Inflammatory Activity by Inhibiting 5-LOX (LTB_4_ Assay)

For the LTB_4_ (Leukotriene B4) test, the products of the arachidonic acid production can be directly measured via HPLC after the enzyme reaction in the thrombocytes and the subsequent activation reaction have taken place. The produced amounts of LTB_4_ and 5-HETE (5-Hydroxyeicosatetraenoic acid) were directly detected at 270 nm, revealing the inhibitory potential of the tested substance (inhibition by zileuton as positive control). For the screening, 1.0 L of cow blood was collected in a plastic bucket that already contained 100 mL of sodium-EDTA solution (0.077 M) as an anticoagulant, immediately after sacrificing the animal. The blood was centrifuged at 200× *g* for 20 min, after which the thrombocytes containing the supernatant were separated. In order to facilitate the lyses of the erythrocytes, the remaining pellet was re-suspended in 800 mL of water. After 30 s of incubation, 400 mL of hypertonic PBS solution was added, followed by another centrifugation step at 485× *g* for 10 min. The resulting cell pellet was re-suspended in 50 mL of isotonic PBS solution and transferred to 20 mL of Histo-Paque (Sigma Aldrich, Taufkirchen, Germany). After yet another centrifugation for 45 min at 675 g, lymphocytes and macrophages that did not sediment in this setup were removed, while the granulocyte pellet was re-suspended in 10 mL of isotonic PBS solution. After an additional washing step in isotonic PBS solution, the granulocytes were re-suspended in O_2_-saturated, isotonic PBS solution, which had been continuously stirred on a magnetic stirrer at maximum speed for 5 min, at an exact cell density of 6 × 10^7^ cells per ml. Although this granulocyte isolation protocol was performed at room temperature, the light exposure of the cells had to be limited as much as possible. For the enzyme reaction, a glass centrifuge tube was filled with 2.5 µL of the *Aloe* constituent test solution, or 2.5 µL of DMSO for the kinetic and control measurements. Subsequently, the tubes were filled with 0.8 mL of the above-described granulocyte suspension and incubated for 5 min at 37 °C in the water bath under constant shaking. Thereafter, 0.2 mL of calcium chloride was added, followed by an additional 5 min of incubation at 37 °C. In the next step, leukotriene production was induced by the addition of 2.5 µL of calcium ionophore buffer to the reaction mixture. After an additional 5 min (this variable was varied, respectively, in the case of the kinetic measurements) of incubation at 37 °C, the enzyme reaction was terminated by adding 1 mL of a solution of acetonitrile/methanol (1:1) that contains NDGA (nordihydroguaiaretic acid) as an antioxidant and PGB_2_ (prostaglandin B2) as an internal standard. All glass centrifuge tubes were put into an ice-water bath immediately after termination of the reaction, sealed, and incubated in the ice-water bath for 20 min. Thereafter, centrifugation was performed for 15 min at 4000 × *g* under constant cooling at 4 °C. The supernatants were filled into vials, which were flanged and stored at −20 °C for further preparation. Each incubation setup contained 4 control and 8 sample measurements. Each defrosted sample was diluted with 10 mL of water and applied to an RP octadecyl extraction column (Macherey-Nagel, Düren, Germany), which had previously been washed successively with 10 mL MeOH, 5 mL water, and 5 mL of a 0.1% EDTA solution. After washing the column two times with 5 mL of water, the adsorbed substances were eluted with 3 mL of MeOH. This eluate was diluted with 3 mL of water. Subsequently, the concentration of LTB_4_ was directly measured by HPLC using a Nucleosil 7 µm C18 column (250 mm × 4.6 mm) (Macherey-Nagel, Düren, Germany) with tetrahydrofuran/MeOH/aqueous EDTH solution (0.1%)/acetic acid (25:30:45:0.1; *v*/*v*), pH 5.5 (conc. NH_4 aq_) as a mobile phase, at a flow rate of 0.9 mL/min. UV detection took place at 270 nm. For measuring the 5-HETE concentration, only the mobile phase was changed to MeOH/H_2_O/acetic acid (77:23:0.1; *v*/*v*), pH 5.5 (conc. NH_4 aq_) with a flow rate of 1.0 mL/min. UV detection took place at 232 nm. The inhibition of the 5-lipoxygenase systems was then calculated from the measured amounts of LTB_4_ and 5-HETE. The relative concentration of LTB_4_/5-HETE in each sample equals the ratio of the peak areas of LTB_4_/5-HETE, relative to the internal standard PGB_2_. The inhibition of the 5-lipoxygenase system, therefore, equals the ratio between the LTB_4_/5-HETE value in the presence and in the absence of the respective *Aloe* constituent.

## Data Availability

The data presented in this study are available in this article.

## Sample Availability

Samples of the compounds are partially available from HWR and RM.

## Abbreviation

CC	Column chromatography
CD	Circular dichroism
COX-1	Cyclooxygenase-1
ESI-MS	Electrospray-ionization mass-spectrometry
HMBC	Heteronuclear multiple-bond correlation spectroscopy
HMQC	Heteronuclear multiple-quantum correlation spectroscopy
HSCCC	High-speed countercurrent chromatography
5-LOX	5-lipoxygenase
LTB4	Leukotriene B4
MDA	Malondialdehyde
